# RG1-VLP and Other L2-Based, Broad-Spectrum HPV Vaccine Candidates

**DOI:** 10.3390/jcm10051044

**Published:** 2021-03-03

**Authors:** Bettina Huber, Joshua Weiyuan Wang, Richard B. S. Roden, Reinhard Kirnbauer

**Affiliations:** 1 Department of Dermatology, Medical University of Vienna, 1090 Vienna, Austria; bettina.huber@meduniwien.ac.at; 2 Department of Pathology, The Johns Hopkins University, Baltimore, MD 21218, USA; jwang150@jhmi.edu (J.W.W.); roden@jhmi.edu (R.B.S.R.); 3 PathoVax LLC, Baltimore, MD 21205, USA; 4 Department of Gynecology and Obstetrics, The Johns Hopkins University, Baltimore, MD 21218, USA; 5 Department of Oncology, The Johns Hopkins University, Baltimore, MD 21218, USA

**Keywords:** human papillomavirus, minor capsid protein L2, RG1-VLP, broad-spectrum prophylactic HPV vaccine

## Abstract

Licensed human papillomavirus (HPV) vaccines contain virus-like particles (VLPs) self-assembled from L1 major-capsid proteins that are remarkably effective prophylactic immunogens. However, the induced type-restricted immune response limits coverage to the included vaccine types, and costly multiplex formulations, restrictive storage and distribution conditions drive the need for next generation HPV vaccines. Vaccine candidates based upon the minor structural protein L2 are particularly promising because conserved N-terminal epitopes induce broadly cross-type neutralizing and protective antibodies. Several strategies to increase the immunological potency of such epitopes are being investigated, including concatemeric multimers, fusion to toll-like receptors ligands or T cell epitopes, as well as immunodominant presentation by different nanoparticle or VLP structures. Several promising L2-based vaccine candidates have reached or will soon enter first-in-man clinical studies. RG1-VLP present the HPV16L2 amino-acid 17–36 conserved neutralization epitope “RG1” repetitively and closely spaced on an immunodominant surface loop of HPV16 L1-VLP and small animal immunizations provide cross-protection against challenge with all medically-significant high-risk and several low-risk HPV types. With a successful current good manufacturing practice (cGMP) campaign and this promising breadth of activity, even encompassing cross-neutralization of several cutaneous HPV types, RG1-VLP are ready for a first-in-human clinical study. This review aims to provide a general overview of these candidates with a special focus on the RG1-VLP vaccine and its road to the clinic.

## 1. Human Papillomaviruses (HPV)

So far, more than 220 human papillomavirus (HPV) genotypes have been identified. This large group of double-stranded DNA viruses is grouped into five genera (alpha, beta, gamma, mu and nu) based on the nucleotide sequence of the major structural protein L1, and can be classified into mucosal or cutaneous types based upon their preferential infection site [1,2]. Generally, HPV encode at least six early genes (E1, E2, E4, E5, E6 and E7) and two late genes (structural L1 major and L2 minor capsid proteins). E1 and E2 are important for viral genome replication and its regulation, E4 promotes virion release from keratinocytes, while oncogenes E6 and E7 interfere with the host’s cell cycle regulators to ensure viral genome replication.

The HPV infection starts by viral binding to heparin sulfate proteoglycan (HSPG) moieties within the epithelial basement membrane (BM) of mucosa or skin exposed by minor abrasion. Attachment triggers a conformational change within the viral capsid that exposes a furin/pro-protein convertase site within the N-terminus of L2 [3]. Upon cleavage, another conformational change exposes the L2 N-terminus with its cross-neutralization epitopes but also uncovers a formerly occluded and still unknown keratinocyte entry receptor(s) within L1. During wound healing, the virus is transferred onto basal keratinocytes migrating in to close the wound, and is thus able to establish an infection in mitotically active cells. After an initial amplification phase, the viral genome is maintained as episome and in low copy numbers (~10^2^) in such epithelial basal stem cells [4]. Viral gene expression is tightly regulated but some oncogenic mucosal HPVs can drive cell cycle progression for genome amplification in the basal and parabasal cells layers, while other types generally promote high level vegetative replication in the mid to upper epithelial layers in association with E4-mediated collapse of keratin bundles. As infected cells undergo terminal differentiation, and L1 and L2 capsid protein expression, genome packaging and viral maturation occurs in the superficial keratinocytes layers. Important for L1 capsid assembly and stabilization is the generation of inter-L1 disulfide bonds in an oxidative environment, which is a slow process occurring during desquamation [5,6]. The viral capsid is a T = 7 non-enveloped icosahedral structure composed of 360 copies of the major structural protein L1 that further assembles into 72 capsomers (or pentamers). This pseudo-symmetry can be upheld because L1 capsomers can occupy both pentavalent and hexavalent positions [7]. In contrast, the minor structural protein L2 is present in varying numbers of 12–72 molecules that appear buried beneath the lumen of L1 capsomers and is only transiently exposed during the entry process [8]. L2 is essential for infectivity, participates in viral genome encapsidation, capsid stability via L1 interaction, endosomal escape and guidance of the viral genome into the host nucleus, but it is not required for capsid assembly (reviewed in [9]). The top of the antiparallel-stranded beta-jellyroll L1 core fold is composed of hypervariable loop structures against which the majority of neutralizing L1 antibodies are directed against [6].

### 1.1. Mucosal HPV

Mucosal HPV can be further grouped into high-risk (hr) or low-risk (lr) types based upon their oncogenic potential, the former being the causative agent of a variety of ano-genital cancers, predominantly cervical cancer (CxCa), but also vaginal-, vulva-, penile-, and anal cancers, and a subset of oro-pharyngeal cancers [10,11]. Around a dozen mucosal HPV types are categorized as carcinogenic (HPV16/18/31/33/35/39/45/51/52/56/58/59), while HPV68, HPV26/53/66/67/70/73/82/30/34/69/85/97 are categorized as probably or possibly carcinogenic, respectively [11]. The majority of HPV types are lr types (including HPV6/11/13/40/43/44/74 etc.), and especially HPV6 and HPV11 cause the majority of genital warts (condylomata acuminate) or the more severe recurrent laryngeal papillomatosis in children. Lesions mediated by lr types are usually self-limiting and very rarely progress to cancer [12]. HPV16 and HPV18 are the two most frequent hr types responsible for ~50% and 20% of all CxCa cases, and a large portion of head and neck cancers appear to be exclusively associated with HPV16 [13]. Mucosal HPVs are sexually transmitted and the cumulative lifetime risk to acquire a genital HPV infection can be as high as ~90% [14]. For HPV16, most infections will resolve spontaneously within 2 years, but a quarter will progress to pre-cancer (CIN3) or worse over the next decade [15]. CxCa is the fourth most common cancer around the world, from which more than a quarter million of women die each year. Such a high number is attributed to CxCa cases particularly in developing countries that cannot afford routine cytological cervical screening nor costly HPV vaccines.

### 1.2. Cutaneous HPV

High risk mucosal HPVs are found within the genus alpha, while cutaneous HPV types are predominantly classified within the genera gamma, mu and nu with only a few exceptions in the alpha genus. Cutaneous HPV types are associated with certain skin pathologies, including HPV types that cause common and palmo–plantar warts predominantly on hands or feet in children and immunosuppressed patients [16]. Even though those warts are benign, they are associated with great morbidity as they can be uncomfortable and recalcitrant, causing considerable costs for treatment. HPV types within the genus beta have been originally identified within skin lesions of patients suffering from the rare genodermatosis epidermodysplasia verruciformis (EV) [17]. EV patients develop persistent, generalized warts with high risks for non-melanoma skin cancer (NMSC) progression particularly on sun-exposed skin areas in midlife. Around 90% of these lesions are positive for beta types HPV5 and HPV8, which are thus regarded potentially oncogenic [18]. Organ transplant recipients (OTRs) show an up to 100-fold increased risk for NMSC development, particularly cutaneous squamous cell carcinoma (cSCC) and basal cell cancers [19,20,21]. The risk is increased in countries with higher sunlight exposure and is dependent upon other co-factors, predominantly UV-radiation and immunosuppressive therapy [22,23]. While the role of beta types in cancer development in EV patients and OTR are established or likely, their role in the general population is much more controversial. Beta HPV can be regarded as skin commensals, with an overall prevalence reaching up to ~90% with increasing age, and even ~45% of babies being infected a few days after birth [24,25,26,27]. Hair follicles have been found to act as a viral reservoir in which infection is being maintained in stem cells in the bulge region [25,27]. Additionally, beta HPVs use different, less rigorous molecular mechanisms than their hr mucosal counterparts in influencing the cell cycle and apoptosis (reviewed in [28]). It is hypothesized that a number of beta HPVs initially act in accordance with the main carcinogen UV-light by maintaining UV-damaged infected cells in a proliferative state to propagate the infection. This is achieved by influencing cell cycle progression, and molecules important in DNA damage repair. Eventually, acquired mutations are able to drive cancerous progression and the viral genome, which is kept episomally in beta HPV infections, is lost. In accordance with such a “hit-and-run” mechanism, precancerous lesions (actinic keratosis) are often found beta HPV-positives while the virus is lost in cSCC [29,30]. In contrast to the case in EV patients, no particular beta type predominates in cSCC in OTRs or the general population, pointing towards the need for a broadly cross-protective HPV vaccine.

## 2. Licensed HPV Vaccines

The first HPV vaccines licensed were bivalent Cervarix^TM^ (GlaxoSmithKline, GSK, Brentford, UK) and quadrivalent Gardasil-4^®^ (Merk and Co, Inc., Kenilworth, NJ, USA) in 2006 that both target around 70% of CxCa mediated by the most frequent hr types HPV16 (~50% of CxCa) and HPV18 (~20% of CxCa). Gardasil-4 additionally includes L1-VLP of the two lr types HPV6 and HPV11, thus protecting against 90% of genital warts (condylomata acuminata). L1-VLP induce a predominantly type-restricted immune response as neutralizing antibodies, which mediate protection, are raised against conformational epitopes within the hypervariable surface loops of the capsid. Some reports suggested a limited ability for cross-protection against phylogenetically related types [31,32]. To increase the breadth of the protective spectrum, Merck developed Gardasil-9^®^, which includes L1-VLP of five additional hr types (HPV31/34/33/52/58) to their Gardasil-4 formulation, thus potentially targeting up to 90% of CxCa. Since the remaining 10% of CxCa are still unaddressed, there is still the need to perform routine cervical screenings (Pap smears) even in vaccinated women. Recently a HPV16/18 L1 VLP vaccine, Cecolin, was approved in China.

These licensed vaccines are produced by recombinant expression of L1 in yeast (Gardasil^®^) or insect cells (Cervarix^TM^) or bacteria (Cecolin), which self-assemble to virus-like particles (VLP) that are morphologically similar to native virions [33,34]. While Gardasil^®^ employs the widely used aluminum salt (amorphous aluminum hydroxyphosphate sulfate, alum) as adjuvant, Cervarix^TM^’s L1-VLP are adjuvanted with ASO4 adjuvant, combining the toll-like receptor 4 agonist 3-O-desacyl-4’-monophosphoryl lipid A (MPL) adsorbed onto aluminumhydroxide (Al(OH)_3_). L1-VLPs have shown to be immunogenic and able to induce high-titers of protective neutralizing antibodies (nAb), which are triggered by repetitive and tightly spaced epitopes that readily activate B cells via the B cell receptor or toll-like receptor cross-linking. Additionally, L1 is rich in T cell epitopes needed to provide B cell help [35,36,37].

Vaccination is recommended before sexual debut prior to exposure with hrHPV as L1-VLP-raised antibodies do not have any effect upon an already existing infection or disease, thus vaccine efficacy decreases with increasing age. Adolescents up to the age of 15 years are recommended to receive two doses, while older teens and adults (up to the age of 45 years) are recommended three doses in order to compensate for lower antibody titers, even though the minimal protective antibody levels are still unknown. In addition, there is evidence than even one immunization is sufficient for protective immunity [38,39]. Antibody titers show the highest level a few weeks after the final boost, with levels decreasing over time and eventually reaching a plateau, which is still several logs higher than antibody levels after a natural infection [40,41]. Long-term clinical studies provided evidence of the induction of an anamnestic response and a long lasting protective antibody response more than a decade after vaccination [42,43,44].

All three vaccines are protein based and do not contain any viral DNA, and have proven safe and immunogenic even in immunosuppressed populations, including HIV-positive children and adults and OTRs, and are recommended in a three-dose regimen in these populations [45,46,47]. L1-VLP-based vaccines do not induce any therapeutic effect and cannot eliminate an established infection as the L1 protein is a predominantly nuclear protein only expressed in upper layers of terminally differentiated keratinocytes, but not in (latently) infected basal cells.

CxCa is the fourth most common cancer in women worldwide, with around 500,000 new cases and ~250,000 deaths per year. Developing countries carry a major CxCa burden because they have not yet been able to routinely implement either cervical screenings or HPV vaccination programs even though some organizations such as GAVI, the Global Vaccine Alliance, have achieved immense success in negotiating a reduced vaccine cost for eligible countries. A part of the high vaccine costs can be attributed to the multivalent and thus very complex formulation, which makes it highly unlikely that L1-VLP of all relevant hrHPV types, yet alone mucosal lr or NMSC-associated cutaneous types, will be included in future L1-based HPV vaccines. Additionally, high costs arise because HPV vaccines are dependent upon an existing cold-chain for transport and storage. They need to be kept at two to eight degrees Celsius, and any temperature mismanagement, including storage at higher temperatures or accidental freezing, can cause decreased immunogenicity, the latter particularly because of the alum adjuvant, which agglomerates upon freezing [48,49].

Although proven highly effective, the above mentioned shortcomings of L1-VLP drive the need to develop enhanced next-generation HPV vaccines that offer: (i) an increased spectrum of protection covering all clinically relevant hr, lr and ideally cutaneous HPV types, (ii) a less complex or ideally monovalent formulation, (iii) cheaper production by, for example, switching to bacterial expression systems, (iv) reduced dosing and/or needle-free administration, (v) reduced costs for storage and distribution by offering cold-chain independence, and (vi) a combined prophylactic and therapeutic approach. 

This review aims to provide a general overview of L2-based broad-spectrum vaccine candidates—particularly RG1-VLP—and their advancement in clinical studies.

## 3. L2-Based Vaccine Candidates

**Figure 1:**
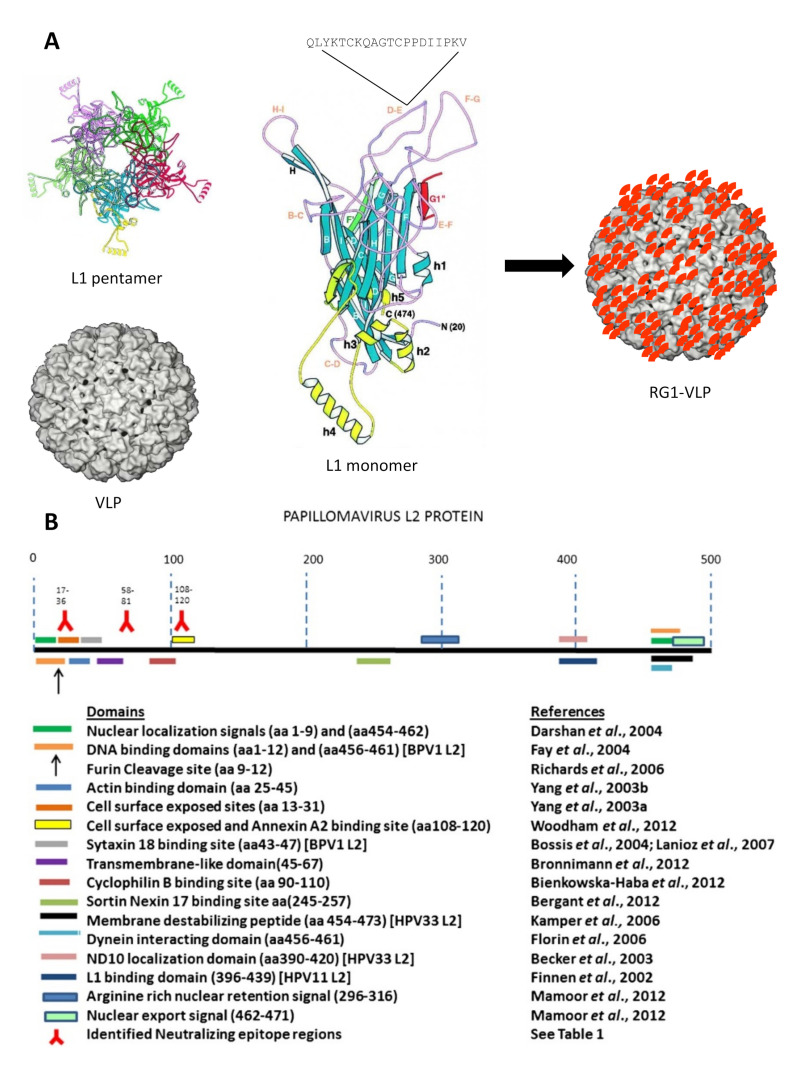
RG1-VLP design (**A**). Five L1 monomers built up a pentamer (capsomer), and 72 capsomers assemble to a virus-like particle. The RG1 epitope is inserted into the HPV16 L1 DE surface loop for presumably 360-fold presentation by the assembled chimeric VLP. Part of the illustration is reprinted from (Chen et al., 2000) with permission from Elsevier. L2 organization (**B**) with N-terminal cross-neutralizing epitopes RG1 aa17–36, aa56–75 and aa108–120 depicted. Reprinted from (Wang and Roden, 2013) with permission from Elsevier.

After natural infection with HPV, around 50% of people mount an L1-specific immune response, but L2-specific antibodies are very rarely found [50]. This likely reflects L2 being buried except during the entry process, after furin cleavage occurs, and its wider spacing, lower occupancy and possibly greater flexibility in the capsid as compared to L1. However, immunizations with L2 protein or peptides alone generate relatively low titers of antibodies, sufficient to protect animals from homologous and even from heterologous papillomavirus infection [51,52]. Thus, even though transient exposure of L2 cross-neutralization epitopes might offer little opportunity for potent antibody response in their natural context, L2-based vaccine candidates are protective against experimental viral challenge [53]. This suggests that low antibody titers are sufficient for protection, which we speculate is because of the slow pace of infection. Cross-neutralization epitopes have been identified exclusively within the first 200 amino (N)-terminal amino acids (aa) of L2 which is highly conserved among diverse papillomaviruses; for example peptides of bovine papillomavirus type 1 (BPV1) L2 aa1–88 [54], BPV4 L2 aa11–200 and aa101–120 [51,55], or HPV16 L2 aa17–36 [56], aa20–38, aa56–75 [57], aa69–81 [58] and aa108–120 [59,60] have shown to induce broadly cross-neutralizing antibodies (Figure 1B).

In vivo, there are distinct mechanisms as to how L1- and L2-raised antibodies can neutralize an HPV infection. L1-raised antibody-mediated protection differs based upon antibody levels. High doses of L1 antibodies prevent viral BM binding leading to the Fc-mediated opsonization of antibody-bound viral particles by phagocytes, mainly neutrophils [61]. In contrast, low L1 antibody levels allow some level of BM binding but potently inhibit L1 engagement of the yet unknown secondary keratinocyte entry receptor causing loss of the virus from the cells. L2 cross-neutralization epitopes become accessible only after virus to BM binding, subsequent capsid conformational changes and furin cleavage. Thus L2-specific antibodies mediate protection by both opsonization and phagocytosis, and by sterically inhibiting stable engagement with the epithelial entry receptor and cell surface. The mechanism as to how systemic L1- or L2-raised antibodies reach the epithelial/mucosal site of infection was shown to be independent upon neonatal Fc receptor-mediated transcytosis (transudation), but instead dependent upon exudation after wounding required for successful infection.

The mouse monoclonal antibody (mAb) RG-1, which recognizes epitope aa17–36 of HPV16 L2, was shown to cross-neutralize HPV16 and HPV18. Furthermore, vaccination with the HPV16 aa17–36 peptide (cross-linked to keyhole limpet hemocyanin (KLH) to provide T cell help) elicits very broadly neutralizing antibodies against mucosal hr and lr HPV6/11/16/18/31/45/52/58, cutaneous beta type HPV5 and BPV1, and confers in vivo protection against homologous experimental HPV16 challenge in mice [56]. Rubio et al. analyzed a panel of mAb raised against HPV16 L2 fused into thioredoxin and found peptide aa20–38 to induce (cross-)neutralization against mucosal hr types HPV16/18/31/45, cutaneous types HPV27/57 and BPV1 [62,63]. Kondo et al. investigated the cross-neutralization potential of several HPV16 L2 N-terminal epitopes against four hr HPV types; the most promising among all the epitopes analyzed was aa56–75 which (cross-)neutralized HPV16/18/31/58 [57]. Kawana et al. raised mAb against HPV16 L1+L2 capsids, identifying aa69–81 of HPV16 L2 as an accessible surface immunodeterminant reacting with human sera positive for multiple hr and lr HPV types [58]. Further, a mAb was mapped to HPV16 L2 aa108–120 that (cross-)neutralized HPV16 and HPV6 [60]. In murine peptide immunizations, the epitope induced serum and vaginal (cross-)neutralizing antibodies to HPV16 and authentic HPV11 virions [59]. In a small placebo-controlled trial, the immunogenicity of this epitope was further investigated in nasal immunization in healthy adults, revealing that a higher antigen dose induced HPV16/52 cross-reactive and cross-neutralizing antibodies in the majority of participants [64]. Importantly, results indicated that the L2 peptide was well tolerated and immunogenic in triggering HPV16 and HPV52 (cross-)neutralizing antibodies, but that the use of a potent adjuvant might have aided in improving induced antibody levels.

All these peptides used for immunizations have in common a promising cross-neutralization capacity. However, the induced serum antibody titers are very low. Thus, several approaches have been employed to overcome L2′s sub-dominance to L1 and stimulate increased cross-neutralization titers. Most often, different scaffolds have been investigated for the improved and more immunogenic presentation of promising HPV16 L2 cross-neutralization epitopes, which include presentation by HPV L1-VLP, non-HPV VLP, or the generation of nanoparticles of concatemeric peptides, filterable aggregates, or fusions of epitopes to immunostimulatory agents (Table 1).

### 3.1. Concatemeric Peptides

To enhance L2’s immunogenicity, Jagu et al. fused different N-terminal L2 regions together to form a multitype concatemeric peptide, which included aa11–88 from five types (HPV1/5/6/16/18), aa11–200 from three types (HPV6/16/18) and aa17–36 from 22 hr, lr and cutaneous HPV types [65,66]. Particularly 11–200x3 and 11–88x5 in combination with potent adjuvants induced high neutralization titers in mice and rabbits that cross-neutralized HPV16/18/31/45/58/6/5, and protected mice from experimental HPV16 challenge four months after vaccination. In another study, Jagu et al. confirmed L2 aa11–88 as a potent cross-neutralization region. Additionally, multimeric fusion proteins comprising L2 aa11–88 from eight HPV types and L2 aa13–47 from 15 HPV types were designed and used in mouse immunizations. Surprisingly, the aa13–47x15 concatemer was less immunogenic than aa11–88x8, which induced cross-neutralization against eleven tested HPV types and provided in vivo protection against vaginal HPV16 challenge [67]. The authors concluded that use of the longer concatemer aa11–88x8 appears to more broadly trigger cross-neutralization because it contains multiple cross-neutralization epitopes when compared to aa13–47, which includes only one cross-neutralization epitope. Both L2 aa11–88x5 and aa11–88x8 protected mice from vaginal challenge with eleven HPV types, including HPV6/16/26/31/33/35/45/51/56/58/59 [68]. This approach is being developed by Bravovax (Wuhan, China). A potential advantage of this system is that the concatemers are produced in bacteria (*Escherichia coli*) and production is thus cheaper when compared to L1-VLP expression in insect cells (Cervarix^TM^) or yeast (Gardasil^®^).

### 3.2. VLP-Based L2-Approaches

Another approach to enhance the immune presentation of L2 epitopes is by insertion within, or conjugation to surface loops of highly immunogenic scaffolds like VLPs from divergent viruses, including bacteriophages or adeno-associated virus. 

Tumban et al. followed up on a finding that the display of foreign epitopes, including HPV16 L2 aa17–31, on RNA bacteriophage PP7 elicits potent anti-L2 antibodies able to (cross-)protect mice against vaginal HPV16 or HPV45 pseudovirion (PsV) challenge [69]. In a study to develop a pan-HPV vaccine, aa17–31 peptides of multiple mucosal and cutaneous HPV types were inserted into the AB surface loop of the PP7 coat protein [70]. All seven tested recombinant fusion proteins, including HPV5/8, HPV6, HPV11/33, HPV16/73, HPV18, HPV45/39 and HPV52/58 L2 PP7 VLP were immunogenic and elicited L2-raised cross-reactive IgG antibodies as measured by L2 peptide ELISA. Even though HPV16 or HPV18 L2 PP7 VLP protected mice from vaginal challenge with HPV16 and HPV18, mice immunized with all seven recombinant PP7 VLP showed the broadest cross-reactivity to L2 peptides as well as cross-protection against vaginal challenge using PsVs of eight different HPV types, including HPV31 that was not included in the VLP mix, and cutaneous HPV5 challenge. Importantly, it was shown that the induced antibody response was long-lived; even though antibody levels declined starting 17 months after mixed L2 PP7 VLP vaccination, mice were still protected against vaginal HPV16/31/45 challenge [71]. Presentation of the HPV16 L2 aa17–31 epitope by N-terminal insertion into the coat protein of another bacteriophage, MS2, induced an even broader cross-reactivity measured by peptide ELISA and in vivo cross-protection against vaginal challenge with nine heterologous HPV types, as well as intradermal HPV5 challenge, when compared to the response mediated by PP7 VLP presenting the same epitope [72]. Such 16L2(17–31) N-term MS2 VLPs were further enhanced by spray-drying the VLPs to a thermostable powder formulation that remained immunogenic and able to induce cross-neutralizing antibodies even after prolonged incubation at 37 °C. Importantly, thermostable dry-powder VLPs stored at 37 °C for 14 months or stored at room temperature for 34 months elicited a protective response against HPV16 or heterologous HPV PsV challenge in mice [73,74,75]. This could greatly simplify vaccination in low resource settings. In another study, it was found that a consensus L2 aa65–85 sequence originating from several hr and lr HPV types induces a more effective cross-neutralization response than the same epitope from the unique types [76]. Bacteriophage VLPs can be produced simply and at low cost in *E. coli*. Unfortunately, the development of this HPV vaccine technology by Agilvax (Houston, TX, USA) is currently on hold after cGMP development.

Nieto et al. and Jagu et al. investigated the presentation of L2 epitopes by adeno-associated virus 2 VLPs (AAVLPs) by genetically inserting HPV16 and HPV31 aa17–31 into separate loops of the VP3 protein of AAV2. AAVLP(HPV16/31L2) immunization using adjuvants triggered robust cross-neutralization in mice or rabbits against HPV16/31/18/45/52/58 and BPV, providing protection against vaginal HPV16 PsV challenge in mice [77]. Importantly, recombinant VLPs retained their immunogenicity even after lyophilization. Additionally, AAVLP(HPV16/31L2)-immunized rabbits were protected from concurrent cutaneous challenges with HPV16/31/35/39/45/58/59 quasivirions (QVs) or native cottontail rabbit papillomavirus (CRPV) virions six- or 12 months post immunization [78]. A phase I study of this vaccine candidate has just been completed by 2A Pharma (Aalborg, Denmark) and results are highly anticipated (NCT03929172).

The presentation of HPV16 L2 aa12–41 by the adenovirus 5 major antigenic capsid protein hexon has been investigated by Wu et al. In mice, immunization with recombinant L2-Adenoviruses induced (cross-)neutralization to HPV16 and HPV73, and in vivo protection against HPV16 vaginal and cutaneous challenge but failed to cross-protect against HPV56. A greater breadth of protection was achieved by display of concatamers of L2 epitopes of multiple HPV types by the adenovirus type 35 protein IX. A mix of such pIX-L2 recombinant adenoviruses presenting the S-fragment (aa11–40 for HPV16) of HPV6, 31, 33 and 16 or HPV11, 52/58, 45 and 18, which spans the RG1 epitope, induced (cross-)neutralizing antibodies against HPV16, 18, 31 and 59. 

The presentation of L2 epitopes by L1-VLP is also a promising strategy to boost L2-raised titers in addition to maintaining the high-titer L1 scaffold-mediated antibody response against the homologous type. We inserted the HPV16 RG1 epitope into the DE surface loop of HPV16 VLP, generating a highly immunogenic chimeric VLP vaccine candidate (see chapter RG1-VLP below) [79,80]. Similarly, RG1 epitope homologs of other mucosal hr and cutaneous HPV types have shown to be immunogenic as well. Cross-protection against four of five alpha-7 mucosal hr HPV types was seen in mice after the passive transfer of mouse sera raised against HPV18 L1-VLP presenting the RG1 homolog from HPV45 [81]. In order to target cutaneous HPV more directly, the RG1 epitope homolog of beta type HPV17 and HPV5 L2 aa53–72 (a homolog to the HPV16 L2 aa56–75 cross-neutralization epitope) was similarly inserted into HPV5, 16 or 18 L1-VLP [82]. The HPV17 RG1-VLP homolog, but not that displaying the HPV5 L2 aa53–72 epitope, induced L2-mediated cross-neutralization to several tested beta HPV types in vitro and protected mice from experimental challenge with PsVs of several beta HPV types. Among the tested L1-scaffolds, HPV16 L1-VLP appeared to most potently present the inserted epitope when compared to insertion in the homologous site in HPV5 L1-VLP. Additionally, the HPV4 RG1 epitope homolog presented on HPV1 L1-VLP induced cross-neutralization and cross-protection against vaginal HPV4 challenge.

Boxus et al. investigated the single or combined insertion of different L2 epitopes both in the DE loop and C-terminus of either HPV16 or HPV18 L1-VLP [83]. Among the tested recombinant VLPs, HPV18 L1-VLP presenting the HPV33 RG1 epitope within the DE surface loop and HPV58 L2 aa56–75 in another loop near the C-terminus, induced cross-neutralization. These immune sera neutralized HPV18/5/6/11/16/31/31/33/45/52/58 and (cross-)protected mice from vaginal PsV challenge with HPV16/11/35/58/45/59 one or six months post vaccination. In addition, rabbits immunized with the double-chimeric L1/L2 VLP were (cross-)protected against papilloma development after infection with HPV18/11/58 QV. The cross-protective efficacy was further enhanced by combinatory vaccination of double-chimeric L1/L2 VLPs together with HPV16/18 L1-VLPs. 

Besides expression in bacteria, another approach for more affordable vaccine production is in plants. Pineo et al. investigated chimeric L1-L2 VLP expression in plants that offers great scalability and rapid production of high yields of antigen [84]. Various L2 cross-neutralization epitopes presented by HPV16 L1-VLP were transiently expressed by Agrobacterium in Nicotiana benthamiana, and particularly L2 aa108–120 L1-VLPs induced limited cross-neutralization against HPV16 and 52 but not against other tested types.

### 3.3. Bacterial Presentation of L2

As an approach to develop a mucosal vaccine candidate administered orally, Yoon et al. generated recombinant Lactobacillus casei (L. casei) that presents the N-terminal HPV16 L2 on its surface [85]. Orally administered lyophilized L. casei-L2 induced HPV16/18/45/58 (cross-) neutralizing antibodies capable to (cross-)protect mice against vaginal challenge with PsVs of these types. 

### 3.4. L2 Fusion to Immunostimulatory Molecules

Another approach to enhance L2-raised responses is linkage to immunostimulatory agents. Alphs et al. linked the HPV16 L2 aa16–37 to the T helper cell epitope P25 and the toll-like receptor (TLR) ligand dipalitoyl-S-glyceryl cysteine (P2C) [86]. The lipoprotein induced potent (cross-) neutralizing antibodies against HPV16/18/45, beta type HPV5 and BPV1 after subcutaneous or intranasal administration, and protected mice from HPV16 and HPV45 PsV challenge at the vaginal or a cutaneous site. 

Rubio et al. investigated the presentation of multiple L2 N-terminal peptides in mono- or multipeptide form presented by bacterial thioredoxin (trx) [62]. All tested candidates were immunogenic in mice, and higher antibody titers were induced by multipeptide presentation. In particular, Trx-L2(20–38) appeared most effective in inducing (cross-) neutralization against HPV16/18/58/45/31. Even more advantageous is the use of a thermostable archaebacterial thioredoxin from Pyrococcus furiosus (Pf) as scaffold to present the highly immunogenic tripeptide form of L2 aa20–38 [87]. Building upon that finding, Seitz et al. investigated the cross-protective potential of a trivalent PfTrx-L2 vaccine candidate [88]. The epitopes aa20–38 from HPV16/31/51 were presented as a polypeptide by PfTrx and induced cross-neutralizing antibodies against 12 of 13 tested mucosal hr HPV types (HPV16/18/45/31/33/52/58/35/59/51/39/68 but not HPV56) in mouse immunizations, and a similar cross-neutralization response was seen in guinea pig immunizations as well. Further, the passive transfer of trivalent PfTrx-L2 mix-raised sera, or active immunization, provided cross-protection against vaginal challenge with tested types HPV16/31/51/18/33. The PfTrx-L2 vaccine candidate is currently being prepared for a first phase I clinical study. In order to further enhance a PfTrx-L2 vaccine candidate, Spagnoli et al. fused L2 aa20–31 of eight types HPV16/18/31/33/35/6/51/59 to PfTrx and the heptamerizing coiled–coil polypeptide OVX313. The resulting PfTrx-L2(8x)-OVX313 appeared thermally stable and induced enhanced cross-neutralizing antibodies against HPV16/18/31/33/35/39/45/51/58 in mice, when compared to the response raised against the monovalent or trivalent PfTrx-L2 vaccine candidates. Additionally, Pouyanfard et al. showed that a thioredoxin-based single peptide vaccine candidate presenting a L2 polytop made up of eight different HPV types and fusion to the OVX313 heptamerization domain induced robust cross-neutralization against a large panel of different mucosal hr and lr types of HPV6/11 in both mice and guinea pigs [89]. The passive transfer of PfTrx-8mer-OVX313-raised mouse or guinea pig serum protected naïve mice from vaginal challenge with PsV from HPV16/18/31/33/35/45/58, and HPV39/56/6/11, respectively. 

Another promising approach uses L2 epitope fusion to the toll-like receptor 5 (TLR 5) ligand flagellin generating a self-adjuvanting antigen expressed in bacteria. Kalnin et al. investigated flagellin fusions with L2 aa11–200, aa11–88x5 or aa11–88x11 that have previously shown to induce cross-neutralization [65,90]. Vaccination in mice verified the induction of (cross-)neutralizing antibodies to HPV16/18, and the vaccine efficacy was evaluated in two pre-clinical settings showing that particularly Fla-L2aa11–88x5 cross-protected mice from vaginal challenge with PsV from HPV16/33/35/56 and prevented HPV6/16/18/31/45/58 QV-mediated papilloma development in a CRPV–QV animal model. In another study, the importance of the RG1 epitope was underlined as incorporation of L2 aa17–38 of five HPV types fused to L2 aa11–200 or aa11–88 enhanced the cross-protective efficacy even further by [91]. Similarly, the generation of a fusion protein, in which the RG1 epitopes of four HPV types, HPV16 L2 aa11–88 and an aa65–85 consensus epitope was fused to flagellin (Fla-5PcL2), induced (cross-)neutralizing antibodies against HPV16/18/31/33/58 in sera and mucosal fluids [92]. Importantly, subcutaneous or intranasal Fla-5PcL2 immunization protected mice from vaginal PsV challenge of HPV39/58/5.

### 3.5. L2-Based Prophylactic and Therapeutic Vaccine Combinations

Generally, L1- and L2-based vaccine strategies are not expected to have a therapeutic effect upon already established HPV infection or induced disease, since structural proteins are expressed in superficial layers of differentiated keratinocytes shortly prior to desquamation. Thus, the immune response to capsid proteins does not target infected basal cells with latent infection. Early proteins have been the targets of various therapeutic strategies, such as E6 and E7 oncogenes that are essential for transformation, and which the expression of cancerous cells are dependent upon for viability. 

A recently conducted pre-clinical study followed up on the thioredoxin-L2-OVX313 fusion vaccine candidate [93]. Zhao et al. fused the HPV16 L2 aa20–31 8mer polytope (3x) and an E7-specific cytotoxic T lymphocyte (CTL) epitope to the thermostable thioredoxin surface that together with the OVX313 heptamerization module assembled into a nanoparticle format [94]. In mouse immunizations, PfTrx-8mer-flank E7-OVX313 induced both a humoral immune response to L2 that protected mice from vaginal challenge with PsVs from HPV11 and 39, and triggered an E7-raised T cell response that protected mice from tumor development in a double challenge of E7-mediated carcinogenesis. 

A further vaccine candidate, tissue antigen–cervical intraepithelial neoplasia (TA-CIN), is a fusion protein of HPV16 L2, E6 and E7 that forms a filterable aggregate that is capable of inducing CTL, T helper responses and antibodies. When administered prophylactically, mice were protected from tumor development following challenge, but the fusion protein also prevented tumor outgrowth when administered therapeutically after tumor induction [95]. TA-CIN has since been investigated in several clinical Phase I and II studies by Cantab/Xenova (Cambridge, UK), verifying the induction of a humoral response as well as a HPV16-specific T cell response in healthy volunteers [96]. The fusion protein was also analyzed together with TA-HPV, a live recombinant vaccinia virus encoding HPV16/18 E6 and E7 protein, in patients with HPV16-positive vulval intraepithelial neoplasia (VIN) [97]. A TA-CIN-mediated effect regarding T cell proliferation and antibody induction was seen, however, there was no marked improvement regarding the clinical symptoms. Another phase II study analyzed TA-CIN immunization after imiquimod topical treatment in VIN grade II and II patients. By histology and HPV testing, over 60% of participants showed HPV16 clearance and around 80% were symptom free. Clearance was associated with lesional CD4 and CD8 infiltrations in contrast to infiltrations of regulatory T cells seen in non-responders [98]. Further, an L2-specific weakly cross-neutralizing antibody response was detected in a subset of TA-CIN vaccinated patients, confirming the potential of TA-CIN as a prophylactic vaccine that, however, might benefit from combined administration with a potent adjuvant [99]. Accordingly, mice vaccinated with TA-CIN together with the saponin derivate GPI-0100 induced high-titer HPV16-neutralizing antibodies as well as HPV31 and HPV58 cross-neutralizing antibodies. Weak cross-neutralization was also seen with other heterologous mucosal HPV types. Importantly, TA-CIN vaccinated mice were protected from cutaneous HPV16 PsV challenge and tumor growth in a TC-1 tumor challenge. Similarly, vaccination in macaques elicited both a HPV16 neutralizing and a weakly cross-neutralizing antibody response, as well as E6- and E7-specific antibodies and IFN-γ producing T cells [100]. A safety and feasibility study in HPV16-positive CxCa patients is currently underway (NCT02405221).

A similar approach has been taken by Cantab to develop a therapeutic vaccine against genital warts-causing HPV6. Tissue antigen–genital warts (TA-GW) is a fusion protein of HPV6 E7 and L2 thus designed to stimulate cellular- and humoral immunity necessary to cause the regression of existing and prevent recurrence of genital warts. The safety and immunogenicity of TA-GW adjuvanted with Alhydrogel^®^ was investigated in two clinical studies revealing the induction of T cell responses as well as a humoral response against L2 and E6. However, a clear effect of TA-GW vaccination could not be established since spontaneous regression of genital warts (GWs) at similar rates is common [101,102,103].

Another approach, which is currently being investigated in a clinical study, is a DNA vaccine that linked the HPV16 early proteins E6 and E7, as well as L2, to calreticulin (CRT) (NCT03913117; NCT04131413) [104]. Naked DNA vaccines offer advantages like large-scale production, safety and efficient delivery of DNA to dendritic cells that effectively trigger CD4+ and CD8+ responses. The pre-clinical data are promising, since vaccination with hCRTE6E7L2 in mice was shown to induce E6- and E7-specific CD8+ cells that protected mice in a TC-1-mediated tumor protection experiment, and an L2-specific protective antibody response against HPV16 was demonstrated. Further, pNGVL4a-hCRTE6E7L2 DNA electroporation has shown therapeutic effects in mice carrying a vaginal E6- and E7-expressing tumor [105]. CD8 T cell induction to early proteins that mediate a therapeutic effect was seen in CD4-depleted mice as well, providing first evidence for potential vaccination in HIV+ patients or organ transplant recipients.

## 4. RG1-VLP

### 4.1. Pre-Clinical Data

Our approach to increase the breadth of protection mediated by L2′s cross-neutralization potential uses HPV16 L1-VLP as a scaffold to present the RG1 epitope from an immunodominant surface loop [80]. Genetic insertion within the HPV16 L1 DE loop displays the RG-1 in a densely packed and highly repetitive fashion, presumably 360 times, on the VLP surface (Figure 1A). In mouse and rabbit immunizations, 16L1-16L2aa17–36 (termed RG1-VLP), combined with alum plus monophosphoryl-Lipid A (MPL) adjuvants, induced (cross-) neutralizing antibodies against HPV6/11/16/18/31/45/52/58 and cutaneous beta type HPV5. In an extensive pre-clinical study, reproducibility of a cross-neutralizing antibody response to mucosal hr types HPV16/18/45/31/33/52/58/35/39/51/59/68/73/26/69/34/70, lr types HPV6/11/32/40 and cutaneous types HPV2/27/3/76 was confirmed in additional rabbit immunizations by native virion- and PsV-based neutralization assays. Importantly, immune sera protected mice from experimental vaginal challenge using PsVs of hr types HPV16/18/45/31/33/52/58/35/39/51/59/68/56/73/26/53/66/34 and lr types HPV6/43/44, which cover ~96% of all CxCa. Immune sera with undetectable cross-neutralizing antibodies to HPV58 by the standard PsV-based neutralization assay did confer cross-protection against this type in vivo, indicating that even lower cross-neutralization titers might provide sufficient protection. RG1-VLP were shown to induce B cell memory as a booster immunization raised diminished cross-neutralization titers back to their former levels. In a pre-clinical dose finding study, three doses of 25 µg of RG1-VLP in rabbits induced a similar cross-neutralizing antibody response to tested types HPV16/18/31/52/45/33/58/26/70 as 125 µg of RG1-VLP, indicating dose-depending saturation of the RG1 epitope response [106]. Additionally, a low dose (5 µg) of RG1-VLPs was shown to induce similar levels of HPV16-raised neutralizing antibody titers when compared to the response to 1/4th of a dose of Cervarix^TM^. Importantly, it was shown that two doses of 5 µg of RG1-VLP are able to trigger cross-neutralizing antibodies against HPV16/18/33/58/26/20 as well, albeit at lower titers.

Based upon these encouraging findings, RG1-VLP have been produced under cGMP sponsored by the US National Cancer Institute’s (NCI) PREVENT Cancer program [107] for a first in human multicenter phase I study scheduled to start in 2021.

A sustained cold-chain is an important requirement to preserve HPV vaccine antigenicity and a significant bottleneck limiting worldwide distribution. For licensed vaccines Cervarix^TM^ and Gardasil-9, the recommended temperature range for vaccine storage is narrow (2–8 °C without freezing). When exposed to elevated temperatures, the disintegration of VLP content can result in loss of antigenicity and vaccine efficacy. 

To facilitate storage and transportation, we have initiated studies to increase RG1-VLP thermostability by exploring lyophilization conditions of RG1-VLP in the presence of an alum adjuvant. However, a required first step in lyophilization is often freezing, which cannot be employed by alum-adjuvanted vaccines due to agglomeration. HPV16 L1 capsomers have been embedded within organic glasses built up by trehalose during lyophilization, which has rendered capsomers immunogenic and thermostable even after incubation at 50 °C for 12 weeks [108]. In ongoing studies, RG1-VLPs were lyophilized by encasing the antigen–alum mix in a sugar matrix. This procedure revealed preparations that resisted high temperatures up to 70 °C for one month without impairment of antigenicity, a competitive advantage over licensed HPV vaccines. Immunizations of mice induced (cross-)neutralization of several tested hr, lr and cutaneous HPV types (Huber B, Garcea R, Roden R, Kirnbauer R, unpublished), suggesting a possible broad spectrum of protection provided by this thermostable HPV vaccine candidate.

### 4.2. Challenges of an L2-Based Vaccine Candidate

Although L2 appears to be promising target to develop a broad-spectrum HPV vaccine, the strength and longevity of L2-raised immune responses need to be considered. L2-induced (cross-)neutralizing antibody titers are generally several logs lower than type-restricted titers induced by homologous L1-VLP. Even if L2 is presented by highly immunogenic viral capsids as scaffold, this raises the question of durability of induced cross-neutralization responses. Importantly, RG1-VLP and L2-PP7 VLP studies have shown that even low L2-specific antibody titers provide (cross-)protection against experimental animal challenge for at least one year post immunization [71,79]. Further, the induction of B cell memory was confirmed for RG1-VLP vaccination, since a boost by 1 year raised antibody titers to initial levels. Nevertheless, the longevity of L2-raised protective responses needs to be further analyzed given long-term protection provided by licensed vaccines. 

We have sought to address this duration of protection question without the need of additional boosters. Utilizing RG1-VLP particles from engineering runs from the NCI PREVENT program, several recent in vivo studies in both mice and rabbits were performed [107]. For example, utilizing the cotton tail rabbit papillomavirus (CRPV) model, in a one year long head-to-head vaccine study against Gardasil-9, it was shown that vaccination with RG1-VLPs adjuvanted with aluminum hydroxide alone (Alhydrogel^TM^) provided complete in vivo protection against nine different HPVs (HPV6,16,31,45,52,58,35,39,59). Importantly, in vivo protection lasted for one year without additional boost and was retained to HPV35, 39 and 59. In contrast, Gardasil-9 was unable to protect against these non-vaccine HPV types as expected due to type-restriction afforded by L1-VLP vaccines. Utilizing ELISA assays, durable although lower L2-specific titers were also detected to these different HPV RG1s. Interestingly, there was no difference between the HPV16 L1-VLP titers between Gardasil-9 or RG1-VLP, demonstrating that incorporation of the RG1 epitope into the HPV16 VLP platform does not affect induction of L1-type restricted high-titer neutralizing antibodies. 

To improve the immunogenicity of L2 titers, the addition of an adjuvant may be useful. In a recent study by Zacharia et al., RG1-VLPs were evaluated in two doses formulated with Alhydrogel^TM^ or in combination with a bacterial enzymatic combinatorial chemistry (BECC)-derived toll-like receptor 4 (TLR 4) agonist [109]. Results indicated that adjuvanting with BECC/Alhydrogel allowed for 75% reduction in antigen dose while still retaining equivalent magnitudes of responses to the full RG1-VLP dose with Alhydrogel. Collectively, these studies show that optimization of the RG1-VLP formulation can result in longer-lasting humoral immunity and at a lower dose amount.

L2-based vaccine candidates promise possible advantages beyond expanding protection to a larger spectrum than technically feasible by multivalent L1-VLP vaccines. Due to their mono-valency, production costs are expected to be cheaper compared to licensed multivalent HPV L1-VLP vaccines enabling prime-boost vaccinations in economically disadvantaged regions.

A sustained cold-chain is an important requirement to preserve HPV vaccine antigenicity and a significant bottleneck limiting distribution. Similar to many other vaccines, refrigeration is essential to prevent vaccine degradation and ensure vaccine potency, which is a critical feature, especially in developing countries with lesser infrastructure. Several L2-based vaccine approaches aim to provide for thermostable formulations either by lyophilization or spray-drying to counteract cold-chain limitations (see Table 1).

A final challenge for L2-based vaccine candidates would be the design of clinical studies providing evidence for non-inferiority to licensed HPV vaccines regarding the level of protection to vaccine-included HPV types, particularly HPV16 and HPV18. Especially for platforms using L2 antigens alone, because the neutralizing antibodies produced are fundamentally different, it is anticipated that such vaccines will have to prove clinical efficacy against pre-cancerous lesions mediated by rarer hr types accounting for 1%–2% of CxCa cases, and thus need to enroll large cohorts or present evidence that infection is prevented via surrogate viral endpoints such as hrHPV DNA detection [110,111]. 

To this end, a chimeric VLP approach like the RG1-VLP may be able to sidestep some of these difficulties and attain approval for non-inferiority via L1-specific neutralizing antibodies, since this vaccine candidate also produces HPV16 L1-VLP specific titers. Indeed, the use of immunogenicity outcomes for the RG1-VLP provides a sound basis for assessing equivalence of protection against types that are targeted in the current standard of care vaccines that would be used as the controls.

## 5. Conclusions

Multiple approaches have been employed to generate L2-based HPV vaccine candidates, and several provide promising results regarding breadth of protection, a durable immunity, immunogenicity after reduced dosing, and thermostability. In contrast to licensed multivalent L1-VLP vaccines, L2-based candidates nearing or in first clinical studies are often monovalent and thus simpler to produce, and aim to overcome shortcomings associated with first-generation HPV vaccines regarding type-restricted efficacy, or cold-chain dependency hindering distribution in developing countries that carry the majority of the CxCa burden. L2-based vaccination strategies have the potential to overcome existing barriers regarding HPV type coverage and global vaccination implementation, aiming to eradicate HPV-associated cancers. 

## Figures and Tables

**Table 1:** List of L2-based HPV vaccine candidates in preparation for or currently in clinical trials.

Antigen		Properties	Status
VLP-based			
HPV16-L1	RG1-VLP	- In vitro (cross-) neutralization against a large panel of mucosal hr, lr and cutaneous HPV types - In vivo cross-protection of mice against all mucosal hr and multiple tested lr HPV types - Long-lasting immunity after 1 year in animals - Potential for thermostable after lyophilization (unpublished) - SF9 insect cell expression (similar to Cervarix^TM^)	cGMP ^1^ production
Bacteriophage	MS2 VLP-16L2 (aa17–31)	- Cross-neutralizes and cross-protects a panel of tested mucosal hr, lr types and beta type HPV5 - Long-lasting immunity - Thermostable after spry-drying and protective even after year-long storage - Single-shot administration induces a (cross-) neutralizing response - Bacterially expressed (*E. coli*)	cGMP production (on hold)
Adeno-associated virus	AAVLP-HPV	- Induces (cross-)neutralizing antibodies against a panel of tested hr types after a two-dose vaccination regimen - In vivo (cross-)protection against QV-challenge of 7 tested mucosal hr HPV types - Thermostable after lyophilization	Phase I completed
Fusion to immunostimulatory agents			
Thioredoxin	trivalent ^2^ PfTrx-L2	- (Cross-)neutralizes a panel of tested hr HPV types - Shows in vivo (cross-) protection against clinically relevant hr types - Thermostable due to use of an archaebacterial thioredoxin	cGMP production
Multimeric L2 proteins			
	aa11–88x5 (HPV6/16/18/31/39)	- (Cross-)protection against a panel of tested hr types - Neutralized HPV18 native virions - Long lived immunity (> 1 year) - Bacterially expressed	cGMP production
	aa11–88x5 or x8		In process development by Bravovax
L2 peptide			
	aa108–120 (HPV16)	- Nasal inoculation of 13 volunteers with 0.1 mg or 0.5 mg peptide (unadjuvanted) or a placebo control - 4/5 participants (0.5 mg group) showed HPV16/52 cross-neutralization	Phase I completed
Combined prophylactic and therapeutic			
CRTE6E7L2 ^3^ naked DNA vaccine with electroporation		- Pre-clinical: triggers E6- and E7-specific CD8 cells protective against tumor challenge and L2-raised neutralizing antibodies - T cell response maintained in CD4-deficient mice (HIV+ model)	Phase I initiation
TA-CIN ^4^	HPV16 L2-E6-E7 Fusion protein	- Safe in humans - Induces B- and T-cell responses to early proteins and L2-specific neutralizing antibodies - Causes local infiltration of CD4 and CD8 cells - Trend towards clinical efficacy when used together with immunomodulator imiquimod	Phase II completed
TA-GW ^5^	HPV6 L2-E7 Fusion protein	- Safe in humans - Triggers a B- and T-cell response - Causing CD4 local infiltrations - Trend towards clinical efficacy	Phase I-IIb completed

^1^ cGMP—current good manufacturing practice; ^2^ PfTrx—pyrococcus furiosus thioredoxin; ^3^ CRT—calreticulin; ^4^ TA-CIN—tissue antigen–cervical intraepithelial neoplasia; ^5^ TA-GW—tissue antigen–genital warts.

## Data Availability

Data available in a publicly accessible repository.
